# Vitamin D Deficiency Is Associated With Metabolic Risk Factors in Women With Polycystic Ovary Syndrome: A Cross-Sectional Study in Shaanxi China

**DOI:** 10.3389/fendo.2020.00171

**Published:** 2020-03-31

**Authors:** Li Wang, Shulan Lv, Fen Li, Xuewen Yu, E. Bai, Xiaofeng Yang

**Affiliations:** Department of Obstetrics and Gynecology, The First Affiliated Hospital of Xi'an Jiaotong University, Xi'an, China

**Keywords:** vitamin D deficiency, metabolic disturbances, polycystic ovary syndrome, obesity, insulin resistance, Shaanxi China

## Abstract

**Background:** Polycystic ovary syndrome (PCOS) is a common endocrine disorder in women at reproductive age, which is characterized by obesity, hyperandrogenemia, and insulin resistance (IR). This study aimed to investigate the vitamin D status, and analyze the relationship between vitamin D deficiency and metabolic risk factors in PCOS women in Shaanxi China.

**Methods:** A cross-sectional study included 169 women diagnosed with PCOS and 114 control women without PCOS. The serum 25(OH)D and metabolic markers were measured. Vitamin D deficiency was defined as serum 25(OH)D concentration less than 20 ng/mL. The primary outcome was the difference in vitamin D status between the PCOS and control groups, the secondary outcomes were correlations between serum 25(OH)D concentration and metabolic risk factors in women with PCOS.

**Results:** The serum 25(OH)D concentration was significantly lower in women with PCOS than in controls (*P* < 0.05), and the prevalence rates of 25(OH)D deficiency and insufficiency were significantly higher in women with PCOS than in controls (*P* < 0.05). The serum 25(OH)D concentration was significantly lower in PCOS women with obesity or IR than in women without obesity or IR (*P* < 0.05), and the prevalence of 25(OH)D deficiency in PCOS women with obesity or IR was significantly higher than in women without obesity or IR (*P* < 0.05). Serum 25(OH)D concentration was significantly negatively correlated with body mass index (BMI), waist-to-hip ratio (WHR), fasting insulin, homeostasis model assessment of insulin resistance (HOMA-IR), total cholesterol, low-density lipoprotein cholesterol (LDL-C), and high-sensitivity C-reactive protein (hs-CRP) (*P* < 0.05). In comparison, serum 25(OH)D concentration was significantly positively correlated with high-density lipoprotein cholesterol (HDL-C) (*P* < 0.05). Increased BMI and WHR, high levels of fasting insulin, HOMA-IR, total cholesterol, LDL-C and hs-CRP were regarded as risk factors, but high level of HDL-C was considered to be protective factor of vitamin D deficiency in PCOS women.

**Conclusions:** Vitamin D deficiency is prevalent in PCOS women in Shaanxi China, especially in those with obesity and IR. The serum 25(OH)D level was correlated with metabolic risk factors in PCOS women. Multi-center randomized controlled trials with large sample sizes are needed to probe the metabolic effect of vitamin D supplementation in PCOS women.

## Introduction

Polycystic ovary syndrome (PCOS) is a common endocrine disorder in women at reproductive age, the prevalence of PCOS ranging from 9 to 18% (1, 2). Obesity, hyperandrogenemia, and insulin resistance (IR) are prevalent characteristics of PCOS (3). Studies have confirmed that PCOS is related to type 2 diabetes mellitus (T2DM), cardiovascular disease and metabolic syndrome (4, 5). A meta-analysis showed that 30–40% of women diagnosed with PCOS accompany IR and compensatory hyperinsulinaemia, and that ~10% of these women will develop T2DM (6). Furthermore, PCOS women were vulnerable to present with dyslipidaemia, such as higher concentrations of triglycerides (TGs) and low-density lipoprotein cholesterol (LDL-C), and lower high-density lipoprotein cholesterol (HDL-C) than healthy women (7).

It is known that vitamin D regulate the skeletal growth and development and calcium/phosphorus metabolism. Nevertheless, vitamin D also is related to other diseases, including immune disorders (8), T2DM (9), cardiovascular disease (10), infectious diseases, and cancer (11). In recent years, the difference of vitamin D levels between PCOS women and healthy women, the relationship between vitamin D and metabolic factors in PCOS women has remained controversial. Some studies have shown that PCOS women had lower serum 25(OH)D concentration than healthy women, and vitamin D deficiency was associated with homeostasis model assessment of insulin resistance (HOMA-IR), hyperinsulinaemia, dyslipidaemia, and metabolic risk factors in patients with PCOS (12, 13). However, other scholars have found different results (14).

Vitamin D deficiency and PCOS are associated with metabolic disorders, but little is known about vitamin D status in women with PCOS in Shaanxi China. Therefore, this cross-sectional study aims to investigate vitamin D status and analyze the relationship between vitamin D deficiency and metabolic risk factors in women with PCOS.

## Materials and Methods

### Study Design and Participants

This survey was a cross-sectional study. A total of 169 women diagnosed with PCOS were recruited through random sampling from five different communities in the city of Xi'an in Shaanxi, China. Participants were selected from the gynecological clinic and women's health clinic in the First Affiliated Hospital of Xi'an Jiaotong University from January 2018 to May 2019. One hundred and fourteen women without PCOS were selected as controls at the same time. The diagnostic criteria of PCOS we used were the modified Rotterdam criteria (15). Women with thyroid disease, congenital adrenal hyperplasia or autoimmune disease were excluded. The protocol was approved by the First Affiliated Hospital of Xi'an Jiaotong University Institutional Review Board.

### Outcome Measures

A questionnaire was administered to each participant in a face-to-face interview. Basic information was collected, including age, body mass index (BMI), waist-to-hip ratio (WHR), and blood pressure. BMI ≥28.0 kg/m^2^ was defined as obesity (16). All biochemical indicators were tested in our hospital including serum 25-hydroxyvitamin D[25(OH)D], follicle-stimulating hormone (FSH), luteinizing hormone (LH), prolactin (PRL), testosterone (T), estradiol (E_2_), progesterone (P), blood glucose, insulin, blood lipids, and high-sensitivity C-reactive protein (hs-CRP). HOMA-IR was calculated to evaluate insulin resistance (IR). HOMA-IR = [fasting glucose(mmol/L) × fasting insulin(mIU/L)]/22.5. HOMA-IR > 2.5 was defined as IR (17). The serum 25(OH)D levels lower than 20 ng/mL were defined as vitamin D deficiency, and women with a concentration of 20~30 ng/mL were defined as vitamin D insufficiency. Serum 25(OH)D concentration of 30~50 ng/mL were defined as normal (18).

The primary outcome was the difference of vitamin D level between the PCOS and control groups. The secondary outcomes were correlations between serum 25(OH)D concentration and metabolic risk factors in PCOS women.

### Statistical Analysis

Data in this study were analyzed using SPSS version 20.0. The continuous variables are presented as mean ± SD, which were performed by Student's *t*-test or variance analysis. The categorical parameters are displayed as numbers (%), which were analyzed by chi-square test. Linear regression analysis was used to analyze the correlation of 25(OH)D concentration with metabolic parameters. The relationship between vitamin D deficiency and metabolic risk factors were analyzed by multinomial logistic regression analysis. *P* < 0.05 was considered statistically significant.

## Results

### Basic Characteristic of the Studied Groups

Table 1 illustrates the baseline characteristics of women in the two groups. The BMI, WHR, and serum LH and T concentrations were significantly higher in women with PCOS than in controls (*P* < 0.05). No significant difference was found when comparing other baseline characteristics between the two groups (*P* > 0.05).

**Table 1:** Baseline characteristics of women between the two groups.

	**PCOS group (*n* = 169)**	**Control group (*n* = 114)**	***P-value^*a*^***
Age (years)^b^	28.4 ± 8.3	27.2 ± 7.9	0.872
BMI (kg/m^2^)^b^	24.7 ± 6.5	21.8 ± 5.6	0.046
WHR^b^	0.9 ± 0.3	0.7 ± 0.3	0.048
Blood pressure^b^			0.409
Systolic (mm Hg)	115.6 ± 14.6	109.5 ± 13.8	0.122
Diastolic (mm Hg)	81.2 ± 9.3	76.9 ± 8.5	0.346
Outdoor exercise^c^			0.302
Never	51 (30.2%)	28 (24.6%)	
≥1 times daily	118 (69.8%)	86 (75.4%)	
Marital status^c^			0.213
Single	45 (26.6%)	23 (20.2%)	
Married	124 (73.4%)	91 (79.8%)	
Basal concentration^b^			
FSH (mIU/mL)	6.8 ± 1.7	7.4 ± 1.8	0.457
LH (mIU/mL)	12.5 ± 3.2	9.3 ± 2.8	0.032
PRL (ng/mL)	15.4 ± 5.8	13.5 ± 4.2	0.053
E_2_ (pmol/L)	89.1 ± 16.4	79.5 ± 14.1	0.179
T (nmol/L)	1.9 ± 0.8	1.4 ± 0.6	0.042
Time of blood detection^c^			0.701
Spring	36 (21.3%)	29 (25.4%)	
Summer	52 (30.8%)	34 (29.8%)	
Autumn	43 (25.4%)	31 (27.2%)	
Winter	38 (22.5%)	20 (17.5%)	

a *T-test or chi-square test*.

b *Data are presented as mean ± SD*.

c *Data are presented as number(%)*.

### Vitamin D Status Between PCOS Women and Controls

**Figure 1:**
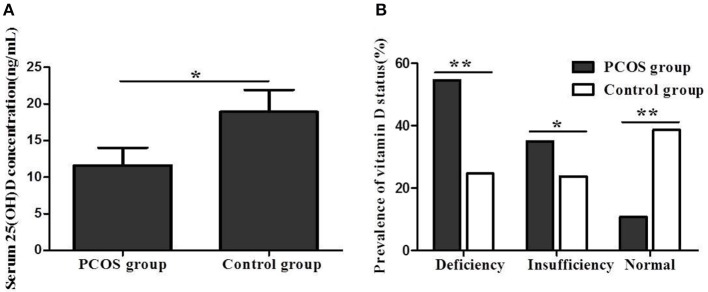
**(A)** Serum 25(OH)D concentration between women with PCOS and control group. **(B)** Vitamin D status between women with PCOS and control group (*P < 0.05, **P < 0.01).

The serum 25(OH)D concentration was significantly lower in PCOS women than in controls (11.6 ± 7.2 vs. 18.9 ± 8.4 ng/mL, *P* < 0.05) (Figure 1A). In addition, the prevalence rates of 25(OH)D deficiency and insufficiency were significantly higher in women with PCOS than in controls (54.4% vs. 37.7%, *P* < 0.01; 34.9% vs. 23.7%, *P* < 0.05). Furthermore, the prevalence of normal 25(OH)D status in women with PCOS was significantly lower than that in controls (10.7% vs. 38.6%, *P* < 0.01) (Figure 1B).

**Figure 2:**
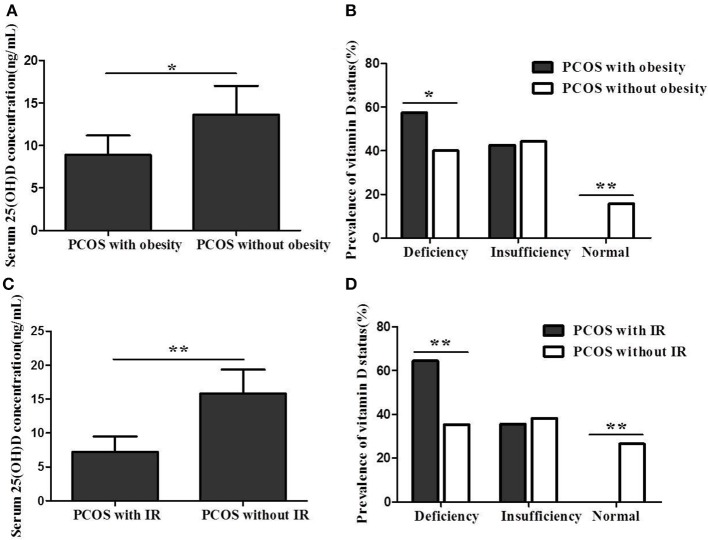
**(A)** Serum 25(OH)D concentration between PCOS women with obesity and women without obesity. **(B)** Vitamin D status between PCOS women with obesity and women without obesity. **(C)** Serum 25(OH)D concentration between PCOS women with IR and women without IR. **(D)** Vitamin D status between PCOS women with IR and women without IR (*P < 0.05, **P < 0.01).

The serum 25(OH)D concentration was significantly lower in PCOS women with obesity or IR than in women without obesity or IR (8.9 ± 3.7 vs. 13.6 ± 5.3 ng/mL, *P* < 0.05; 7.2 ± 2.9 vs. 15.8 ± 4.9 ng/mL, *P* < 0.01) (Figures 2A,C). Additionally, the prevalence of 25(OH)D deficiency in PCOS women with obesity or IR was significantly higher than in women without obesity or IR (57.4% vs. 40.0%, *P* < 0.05; 64.4% vs. 35.3%, *P* < 0.01) (Figures 2B,D).

Table 2 shows the metabolic parameters among different vitamin D statuses in women with PCOS. There were statistically significant differences in BMI, WHR, fasting insulin, HOMA-IR, total cholesterol, LDL-C, HDL-C, and hs-CRP among the three groups (*P* < 0.05). No significant difference was found when comparing fasting glucose and triglycerides among different groups (*P* > 0.05).

**Table 2:** Metabolic parameters among different vitamin D status in women with PCOS.

	**Deficiency group (*n* = 92)**	**Insufficiency group (*n* = 59)**	**Normal group (*n* = 18)**	***P-value^a^***
BMI (kg/m^2^)	27.3 ± 9.2^b^	25.4 ± 8.1	23.5 ± 9.3	0.029
WHR	1.0 ± 0.4^b^	0.9 ± 0.5	0.8 ± 0.3	0.036
Fasting glucose (mmol/L)	5.1 ± 1.9	4.9 ± 1.6	4.8 ± 1.7	0.327
Fasting insulin (mIU/L)	39.6 ± 10.7^b^^c^	33.5 ± 9.9^d^	26.8 ± 8.5	0.012
HOMA-IR	8.9 ± 3.7^b^^c^	7.3 ± 2.8^d^	5.7 ± 2.1	0.009
Total cholesterol (mmol/L)	6.1 ± 1.7^b^^c^	5.5 ± 1.6^d^	4.2 ± 1.4	0.033
LDL-C (mmol/L)	2.5 ± 0.9^b^	2.3 ± 0.8	2.0 ± 0.7	0.041
HDL-C (mmol/L)	1.3 ± 0.6^b^	1.4 ± 0.7^d^	1.8 ± 0.6	0.030
Triglycerides (mmol/L)	2.3 ± 0.9^b^	2.1 ± 0.8^d^	1.9 ± 0.8	0.068
Apo A (g/L)	1.3 ± 0.5	1.4 ± 0.7	1.5 ± 0.3	0.115
Apo B (g/L)	1.0 ± 0.4	0.9 ± 0.3	0.8 ± 0.4	0.632
hs-CRP (mg/L)	2.4 ± 0.9^b^^c^	1.9 ± 0.6^d^	1.4 ± 0.3	0.017

a *Variance analysis*.

b *Deficiency group vs. Normal group, P < 0.05*.

c *Deficiency group vs. Insufficiency group, P < 0.05*.

d *Insufficiency group vs. Normal group, P < 0.05*.

### Vitamin D and Metabolic Risk Factors in PCOS Women

The data in Table 3 suggest that serum 25(OH)D concentration was significantly negatively correlated with BMI, WHR, fasting insulin, HOMA-IR, total cholesterol, LDL-C and hs-CRP (*P* < 0.05). In comparison, serum 25(OH)D concentration was significantly positively correlated with HDL-C (*P* < 0.05). However, no significant correlation was found between serum 25(OH)D concentration and age, fasting glucose, triglycerides, Apo A or Apo B (*P* > 0.05).

**Table 3:** Correlation of serum 25(OH)D concentration with metabolic parameters in PCOS women.

	***R***	***P-value***
Age (years)	−0.089	0.582
BMI (kg/m^2^)	−0.581	0.016
WHR	−0.424	0.032
Fasting glucose (mmol/L)	−0.246	0.237
Fasting insulin (mIU/L)	−0.624	0.003
HOMA–IR	−0.635	0.001
Total cholesterol (mmol/L)	−0.426	0.032
LDL-C (mmol/L)	−0.474	0.022
HDL-C (mmol/L)	0.419	0.043
Triglycerides (mmol/L)	−0.326	0.108
Apo A (g/L)	−0.113	0.567
Apo B (g/L)	−0.076	0.683
hs-CRP (mg/L)	−0.539	0.028

The data in Table 4 suggest that increased BMI and WHR, high levels of fasting insulin, HOMA-IR, total cholesterol, LDL-C and hs-CRP were regarded as risk factors of vitamin D deficiency in women with PCOS. Additionally, outdoor exercise (≥1 times daily) and high level of HDL-C were considered to be protective factors of vitamin D deficiency in PCOS women.

**Table 4:** Multinomial logistic regression model of risk factors for vitamin D deficiency in women with PCOS.

	**95.0% CI**	**OR**	***P-value***
**Age (years)**			
≤ 25	Reference	1.00	–
26–34	0.48–1.91	1.02	0.692
≥35	0.53–2.06	1.13	0.583
**BMI (kg/m**^**2**^**)**			
<25	Reference	1.00	-
≥25	1.23–5.18	2.53	0.011
**WHR**			
<0.85	Reference	1.00	-
≥0.85	1.17–3.92	2.17	0.019
**Hypertension**			
No	Reference	1.00	-
Yes	0.29–2.97	1.24	0.376
**Outdoor exercise**			
Never	Reference	1.00	-
≥1 times daily	0.31–0.93	0.49	0.032
**Fasting glucose (mmol/L)**			
<6.1	Reference	1.00	-
≥6.1	0.57–2.03	1.57	0.119
**Fasting insulin (mIU/L)**			
<20	Reference	1.00	-
≥20	1.96–38.9	5.74	0.008
**HOMA-IR**			
<2.66	Reference	1.00	-
≥2.66	2.58–73.2	10.06	0.003
**Total cholesterol (mmol/L)**			
<5.69	Reference	1.00	-
≥5.69	0.93–2.86	1.98	0.047
**LDL-C (mmol/L)**			
<3.1	Reference	1.00	-
≥3.1	1.88–7.43	2.23	0.035
**HDL-C (mmol/L)**			
<1.55	Reference	1.00	-
≥1.55	0.25–0.89	0.37	0.024
**Triglycerides (mmol/L)**			
<1.47	Reference	1.00	-
≥1.47	1.03–2.96	1.38	0.065
**Apo A (g/L)**			
<1.61	Reference	1.00	-
≥1.61	0.63–2.24	1.48	0.223
**Apo B (g/L)**			
<1.05	Reference	1.00	-
≥1.05	0.56–2.17	1.25	0.446
**hs-CRP (mg/L)**			
<3	Reference	1.00	-
≥3	1.85–6.37	2.18	0.038

## Discussion

More than one billion children and adults are suffering from vitamin D deficiency, which is common worldwide (19). PCOS women manifest a relatively high prevalence of vitamin D deficiency than healthy women, and vitamin D deficiency is associated with ovulatory dysfunction, IR and hyperandrogenism (20). Thus, in recent years, many studies have been performed to analyze vitamin D status of PCOS women (21). Data from our study showed that the serum 25(OH)D concentration was significantly lower, and the prevalence rates of 25(OH)D deficiency and insufficiency were significantly higher in women with PCOS than in controls, the results is similar to previous study (22). It has been confirmed that PCOS women accompany vitamin D deficiency, and 67–85% of them had serum 25(OH)D level <20 ng/mL (23).

Multiple observational studies have shown that low vitamin D concentration is associated with increased BMI, IR, testosterone and dehydroepiandrosterone sulfate (DHEAS) levels in women with PCOS (24), which was consistent with our study, where we showed that serum 25(OH)D concentration was significantly lower in PCOS women with obesity or IR than in women without obesity or IR. In addition, the prevalence of 25(OH)D deficiency in PCOS women with obesity or IR was significantly higher than in women without obesity or IR. Epidemiological studies have confirmed that vitamin D status was negatively associated with diabetes, which may be related to multiple factors. Research confirmed that 25(OH)D can increase the expression of insulin receptors, and inhibit the release of inflammatory cytokines that are proved to cause IR (25). Furthermore, it has been reported that vitamin D has an indirect useful function on insulin role through regulation of extracellular calcium (26). However, other researchers have revealed different results. Ng et al. demonstrated that there was no statistically significant correlation between vitamin D level and BMI, WHR, and metabolic parameters among PCOS women (14). This difference may be explained by differences in sample size, habits of the participants, place of residence, and season of detection of vitamin D.

Concerning the lipid profile, this study demonstrated that serum 25(OH)D concentration in women with PCOS was significantly and negatively correlated with total cholesterol and LDL-C but was significantly positively correlated with HDL-C. This finding was concordant with the results of previous studies (27). It is well-known that cardiovascular disease is associated with high total cholesterol and LDL-C levels and low HDL-C levels. Therefore, vitamin D supplementation may reduce the incidence of cardiovascular disease in vitamin D-deficient women with PCOS by improving dyslipidaemia. However, the relationship between serum 25(OH)D and triglyceride concentration in PCOS women is controversial according to previous studies (28). This study demonstrated that no significant correlation was found between serum 25(OH)D concentration and triglycerides, Apo A or Apo B, which was consistent with the results of Li et al. (12).

Normal metabolism of insulin is affected by activation of inflammatory pathways. According to the published studies, the positive effects of vitamin D on sugar metabolism including regulation of insulin secretion and inhibition of proinflammatory cytokines (29). The data in our study displayed that serum 25(OH)D concentration in women with PCOS was significantly negatively correlated with hs-CRP. Results from a randomized controlled trial showed that vitamin D treatment significantly decreased serum hs-CRP in PCOS women with vitamin D deficiency (30). In addition, serum hs-CRP was improved by high dose vitamin D supplementation among PCOS women with IR (17).

Clinical manifestations of PCOS include obesity, metabolic syndrome and chronic inflammation, which are related to vitamin D deficiency (10–14, 29). Our findings revealed that serum 25(OH)D level in PCOS women was significantly negatively correlated with BMI, WHR, fasting insulin, HOMA-IR, total cholesterol, LDL-C, and hs-CRP. Additionally, serum 25(OH)D concentration was significantly positively correlated with HDL-C. Furthermore, the data from logistic regression model of risk factors for vitamin D deficiency revealed that increased BMI and WHR, high levels of fasting insulin, HOMA-IR, total cholesterol, LDL-C, and hs-CRP were regarded as risk factors of vitamin D deficiency in PCOS women. In comparison, outdoor exercise (≥1 times daily) and high level of HDL-C were considered to be protective factors of vitamin D deficiency in PCOS women. These results suggest that PCOS women with metabolic risk factors are more likely to accompany vitamin D deficiency. Therefore, scholars from various countries have conducted randomized controlled trials on whether vitamin D supplementation can improve the metabolic risk factors of PCOS women. However, the results have been discordant. Vitamin D treatment improved the follicular development, menstrual cycle regulation, insulin resistance, and hyperandrogenism (21). However, Xue et al. showed that vitamin D supplementation did not change the HOMA-IR, LDL, DHEAS, free testosterone, and total testosterone in women with PCOS (31). The different results may be related to sample size, vitamin D supplement dose, participant habits, and place of residence. Therefore, multi-center randomized controlled trials with large sample sizes are needed to explore the metabolic role of vitamin D supplementation in women with PCOS.

This study has some limitations. The sample size of the participants was relatively small. In addition, this was a single-center study of PCOS women in the city of Xi'an in Shaanxi, China. Hence, the results of this research needs to be proved through multi-center surveys with large sample sizes in other areas in China.

## Conclusion

Our data show that vitamin D deficiency is prevalent in PCOS women in Shaanxi China, especially in those with obesity and IR. The serum 25(OH)D concentration was correlated with metabolic risk factors in PCOS women. Multi-center randomized controlled trials with large sample sizes are needed to explore the metabolic role of vitamin D supplementation in women with PCOS.

## Data Availability Statement

All datasets generated for this study are included in the article/supplementary material.

## Ethics Statement

The studies involving human participants were reviewed and approved by The First Affiliated Hospital of Xi'an Jiaotong University Institutional Review Board. The patients/participants provided their written informed consent to participate in this study.

## Author Contributions

LW and XY conceived the study and wrote the manuscript. SL and FL collated data. XY and EB analyzed data. All authors have given final approval of the article to be published.

### Conflict of Interest

The authors declare that the research was conducted in the absence of any commercial or financial relationships that could be construed as a potential conflict of interest.
